# Socioeconomic Inequality in Self-immolation, between Genders; Oaxaca-Blinder Decomposition, Results of Registration-Based Suicide Data

**DOI:** 10.29252/beat-070409

**Published:** 2019-10

**Authors:** Sattar Kikhavani, Yousef Veisani, Fathola Mohamadian, Reza Valizadeh, Ali Delpisheh, Ghobad Moradi, Maryam Bagheri

**Affiliations:** 1 *Department of Psychology, Psychosocial Injuries Research Center, Ilam University of Medical Sciences, Ilam, Iran*; 2 *Psychosocial Injuries Research Center, Ilam University of Medical Sciences, Ilam, Iran*; 3 *Department of Clinical Epidemiology, Ilam University of Medical Sciences, Ilam, Iran*; 4 *Social Determinants of Health Research Center, Research Institute for Health Development, Kurdistan University of Medical Sciences, Sanandaj, Iran.*

**Keywords:** Inequality, Self-immolation, Suicide, Attempts, Decomposition

## Abstract

**Objective:**

To assess the important socio-demographic inequalities in self-immolation in between genders.

**Methods:**

A cross-sectional study, 2011 to 2016, was conducted. A total of 540 completed suicides were recruited to the study. Data were collected by systematic registration suicide data (SRSD) and Legal Medicine Organization (LMO). The concentration index (CI) was used to determine the inequality. The inequality line was decomposed to find out the main sources of inequality in self-immolation between genders by Oaxaca-Blinder approach.

**Results:**

The mean ratio of self-immolation was 21.8% among completed suicides. The decreasing trend was found in self-immolations during 2011-2016 (z = -2.07, *p* = 0.039), the mean rate in six years, was 2.98 per 100.000 populations. Unemployment −.043 (−.07, −0.01), married subject’s −.016 (−.03, −0.00) and low educational level −.005 (−.01, −0.00) were the main inequality source in females compared to males.

**Conclusion:**

Our results suggest that despite the incidence decreasing in self-immolation within 6 years of study period, the inequality was detected in self-immolation. The main socio-demographics in inequalities were lower educational level, married persons and unemployment that prevention programs should be more concentrated in females to a decrease of inequality in self-immolation.

## Introduction

Self-immolation is a fatal method of suicide with adverse consequences [1-3]. Unlike the developed countries and Western societies, self-immolation in developing countries is a common method of suicide [4]. A quarter of completed suicides in Iran are due to self-immolation, consequently, it is a common suicide method in Iran. According to the recent reports in the western provinces, self-immolation is a leading cause of completed suicides in Iran [5]. Some epidemiological aspects of self-immolation in Iran are different from other reigns; the rate of self-immolation is higher in female compared to males. In addition, the rate of mental disorders in self-immolation in Iran is lower than European and North American countries that 96% of attempters suffered from depression and schizophrenia [6].

Self-immolation has a multi factorial nature in our society; socio-demographic factors can be important. Young persons, females, low education and living in rural areas are the main factors that mentioned before [7-9]; however, self-immolation in attempts is one way that they save themselves from internal stress and mental suffering [10]. Hospitalization, vast scars, and disabilities are the common complications of the self-immolation that accompanied by costs of psychosocial rehabilitations [11, 12].

Recently, inequalities in self-immolation in Iran have been shown in 2017 [13], which was conducted based on data in national level. However, regional studies should be conducted to assessing the sources of inequality that are essential for effective and functional prevention programs in the future. In the current study by the exact registration system, we aimed to determine the trend of self-immolation and important socio-demographic sources of inequalities in self-immolation between genders.

## Materials and Methods

*Source of data*

By the cross-sectional study, through of systematic registration suicide data (SRSD) in the six study periods, 2011 to 2016, all of 540 suicide deaths were recruited to the study. Data were collected by official statistics from the Ilam University of Medical Sciences. The institutional review board (IRB) and medical ethics committee approved the study protocol. This information is including; demographic variables, medical history, and other characteristics of attempter. Subsequently, completed suicides were approved by Legal Medicine Organization (LMO). Therefore, SRSD is a valid and up-to-date database towards completed suicides.

*Socio-economic Status*

Based on demographic variables, subjects were stratified to Socioeconomic Status (SES), also advantaged and disadvantaged groups were diagnosed by SES. The predictor variables for grouping subjects to SES were job status, having of personal home, educational level, and income. Principal composition analysis (PCA) analysis was used to stratify of subjects into SES groups according to a Vyas study in 2006 [14].

**Table 1:** Characteristics of completed suicide by self-immolation and other methods

	**self-immolation** **N=118 (Row %)**	**Others** ^a^ **N=422 (Row %)**	**P-Value** ^b^
**Gender**			
**Female**	86 (72.9)	130 (30.8)	<0.001
**Male**	32 (9.9)	292 (69.2)	
**Age**			
<15	0 (0.0)	4 (.95)	0.091
15-24	34 (28.8)	145 (34.3)	
25-44	49 (41.5)	190 (45.0)	
45-64	18 (15.2)	52 (12.3)	
65+	17 (14.4)	31 (7.3)	
**Job**			
Unemployment	16 (13.6)	148 (35.1)	<0.001
Informal work	9 (7.6)	80 (19.0)	
Employee	4 (3.4)	32 (7.6)	
Marketer	4 (7.1)	62 (15.4)	
Housewife	26 (46.4)	291 (72.4)	
Student	10(8.5)	47 (11.1)	
**Marital Status**			
Single	46 (39.0)	212 (50.2)	0.037
Married	67 (56.8)	204 (48.3)	
Beloved	2 (1.7)	4 (0.9)	
Wife died	3 (2.5)	2 (0.5)	
**Place of suicide**			
Home	116 (98.3)	359 (75.6)	0.001
Work place	0 (0.0)	11 (2.6)	
Public place	2 (1.7)	12 (2.8)	
Mountain	0 (0.0)	40 (9.5)	
**Year**			
2011	38 (28.6)	74(18.2)	0.132 3.98
2012	23 (17.3)	68 (16.7)	
2013	20 (15.0)	65 (16.0)	
2014	19 (14.3)	85 (20.9)	
2015	17 (12.8)	67 (16.5)	
2016	16 (12.0)	48 (11.5)	

^a^Fall, medicine, gas, gun, Addictive substances, hanging; ^b^Chi-Square

**Fig. 1:**
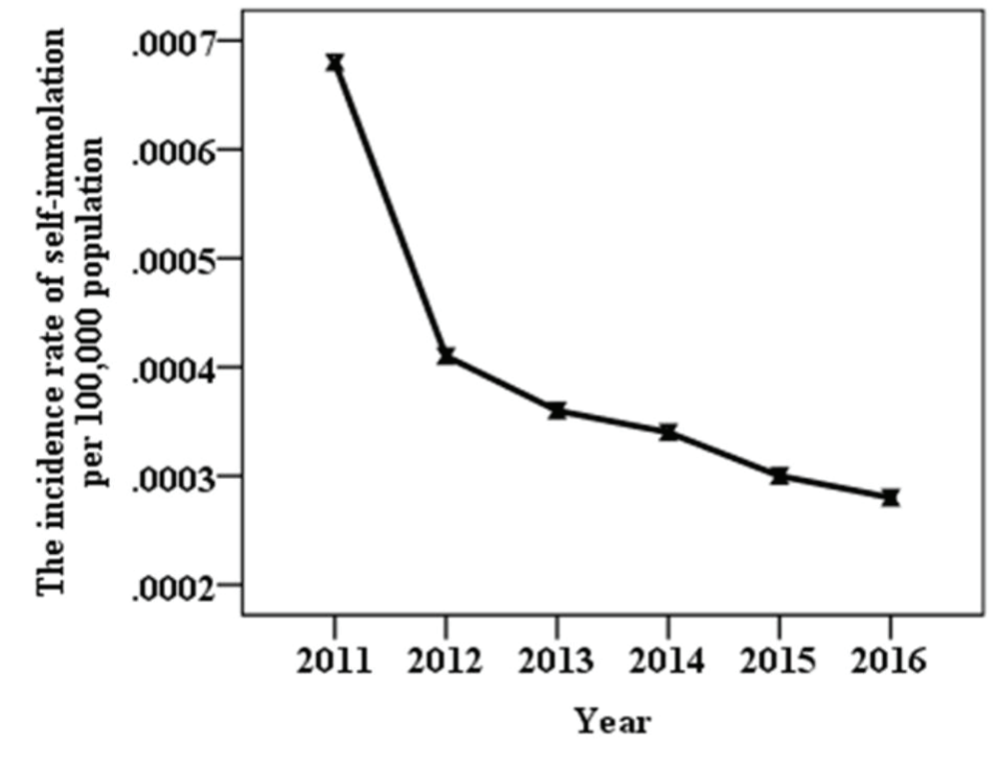
The incidence rate of self-immolation per 100,000 populations, Ilam province, 2011-2016 (by Wilcoxon rank-sum test)

**Table 2:** Pooled results of decomposition in self-immolation by genders

Gender	Coef.	Std. Err.	z	P>z^b^	[95% Conf.	Interval]
						
Differential						
Male^a^	.30	.02	13.3	<0.001	.26	.35
Female^a^	.68	.04	16.7	<0.001	.60	.76
Difference	-.37	.04	-8.0	<0.001	-.46	-.28
Decomposition						
Endowments	-.10	.03	-3.1	0.002	-.16	-.03
Coefficients	-.32	.04	-7.2	<0.001	-.41	-.23
Interaction	.04	.02	1.7	0.080	-.00	.10

^a^Prevalence of self-immolation method; ^b^Chi-Square

**Table 3:** Details of main contributors in self-immolation inequality by genders

**Gender**	**Coef.**	**Std. Err.**	**z**	**P>z** ^b^	**[95% Conf.Interval]**	
Differential						
Male^a^	.30	.02	13.4	<0.001	.26	.35
Female*	.68	.04	16.9	<0.001	.60	.76
Difference	-.37	.04	-8.1	<0.001	-.46	-.28
Explained						
Illiterate	-.005	.006	-0.84	0.400	-.01	.00
Unemployment	-.043	.014	-3.11	0.002	-.07	-.01
Married	-.016	.009	-1.69	0.091	-.03	.00
Urban residence	.002	.003	0.86	0.388	-.00	.00
Total	-.062	.017	-3.49	<0.001	-.09	-.02
Unexplained						
Illiterate	-.000	.027	-0.01	0.991	-.05	.05
Unemployment	-.265	.075	-3.50	<0.001	-.41	-.11
Married	-.017	.047	-0.36	0.717	-.10	.07
Urban residence	.030	.065	0.47	0.640	-.09	.15
_cons	-.061	.118	-0.52	0.604	-.29	.17
Total	-.314	.043	-7.17	<0.001	-.40	-.22

*Statistical analysis*

The incidence rate of self-immolation in this study calculated based on the number of cases per 100,000 populations in Ilam province. Blinder-Oaxaca decomposition determines the partition of inequality between groups by two explained and unexplained components. Explained component shows the main determinants responsible for inequality and diversity between groups. Unexplained component shows differences that cannot explain by determinants that determinants in the underlying logistic regression model. In Stata, the nptrend command was used to test of trend across ordered years 2011-2016 by the Wilcoxon rank-sum test. All *P* values < 0.05 were regarded as statistically significant. Data were analyzed by Stata computer software version 12 (StataCorp, College Station, TX, USA).

## Results

A total of 540 deaths were occurred during 6 years of study. Among 118 (21.8%) was due to self-immolation out of all. A majority of self-immolations were occurred in female (n= 86; 72.9%), 25-44 age groups (49; 41.5%), housewife’s (74; 62.7%), and married cases (67; 56.8%). The mean ratio of self-immolation was 21.8%, while this rate was 28.6 in the first year of study (2011) and 12.0% at the end of study (2016) (Table1).

The trend of self-immolations in 100.000 populations between 2011 and 2016 is shown in Figure 1. The mean rate of self-immolations during six years was 2.98 /100.000 populations, the highest was in 2011 (6.8) and the lowest in 2016 (2.89). Trend analysis was shown that self-immolation rate was decreasing over the time period (z = -2.07, Prob > |z| = 0.039). The decomposing of socio-economic status by Blinder–Oaxaca technique showed that the self-immolation in females was 68% (95% CI: .26, .35%) vs. 30% (95% CI: .60, .76%) in males; the gap of self-immolation between genders was -37% (95% CI: -.46, -.28%). We found that expected change in the prevalence self-immolation in female was 10% due to gender differences, (Table 2). 

According to decomposition results we found that 6% of self-immolation prevalence in female could be decreased if females have had the same characteristics with males, including gender, educational level, marital residence, and job status. Therefore, about 6% of the gap between groups could be explained by differences in gender, educational level, marital and job status and also 31% unexplained with included variables. Unemployment −.04 (95% CI:  −.07, −0.01) was the highest level of contribution in the gaps between genders, other contributors were illiterate −.005 (95% CI:  −.01, −0.00), and married −.01 (95% CI:  −.03, 0.00) (Table 2). 

## Discussion

In this study we aimed to diagnose the main sources of inequality in self-immolation in Ilam province in Iran. Determining the inequality in self-immolation can be an important act that accelerates the strategies that should be conducted to reduce the self-immolation rate in our society. Our study has been achieved based on SRSD and LMO that has registered attempts and completed suicides records since 2010 in Ilam province; therefore, data was up-to-date and reliable. 

The rate of self-immolation was 2.8 /100,000 people in our study population; near to 42% of the rate of self-immolations was dropped during the study period. In one study by Ahmadi in Northern Iran the rate of self-immolation was reported 1/100,000 in general population [15]. Therefore, the incidence rate of self- immolation suicide was different in Iranian population depends of geographic areas. Furthermore, one quarter of all completed suicides was due to self-immolation. In addition, younger ages, females, and married cases had the highest rate of self-immolation. Our findings are consistent with the current systematic review by author name in 2017 which reported that70 % of self-immolations have been occurred in females and in younger ages, with the mean of 27.3 age [3]. Moreover, a systematic review in WHO Eastern Mediterranean Region countries showed that self-immolation was happened 29.4% in females out of all completed suicides in compare with 11.3% in males [16]. 

Analysis of inequality based on genders shows a positive inequality in self-immolation, the prevalence of self-immolation in all 540 suicide deaths in male was 30% vs. 68% in females. Oaxaca-blinder decomposition showed that 10% of inequality could be explained by educational status, job status, marital status, and residence. The important determinant of inequality in self-immolations was unemployment; therefore, each plan to diminish unemployment inequality in genders will lessen the incidence of self-immolation in our society. Therefore, reducing inequality will lead to reduce the amount of self-immolation. However, the precise etiology of higher rates of self-immolations in female compared to males is not clear; however, unequal social supports, and existing prejudices in traditional families can be important in our society [17]. Unfortunately, occasionally, self-immolations in female is a solution to escape from social contradictions, without family support [18].

The second source of inequality in self-immolation between genders was married persons. Our results show that married variable could be explained 1% of inequality in female. this finding have shown in previous in attempters [19]. Some other factors including school concerns, legal/disciplinary problems, family conflict, and mental health conditions such as depression was associated to higher rate of self-immolation in younger females [20]. Consistent to our findings, the higher prevalence of mental disorders has been shown in females in Iran [21]. Housewives are usually busy with home chores and at risk to mental disorders due to lack of sufficient social communications, insurance shortages and lack of economic and social supports. 

It should be mentioned that there were some limitations in our study as fallows. Under reporting of completed suicide due to suicide stigma in our society especially for the young's and females. besides missing data due to imperfection of medical records makes it hard to figure the actual incidence of suicide. Finally, this study is a cross-sectional and the results that derived from the current study should be interpreted with caution.

We can conclude that despite the incidence decrease in self-immolation within 6 years of study period, the inequality was detected in self-immolation. Our results showed the positive inequality in self-immolation between genders, also the main determinant that reduce inequality high to low were unemployment, married subjects and low educational level in females compared to males. According to our results females was the vulnerable groups at risk of self-immolation and the prevention policies should be foxed to these groups in order to reduce the inequality and rate of self-immolation. 
